# Editorial: Advances in Microbial Biofuel Production

**DOI:** 10.3389/fmicb.2021.746216

**Published:** 2021-09-28

**Authors:** Debarati Paul, Anju Arora, Madan L. Verma

**Affiliations:** ^1^Amity Institute of Biotechnology, Amity University Uttar Pradesh, Noida, India; ^2^Division of Microbiology, Centre for Conservation and Utilisation of Blue Green Algae, Indian Agricultural Research Institute, New Delhi, India; ^3^Department of Biotechnology, School of Basic Sciences, Indian Institute of Information Technology, Una, India

**Keywords:** biofuel, advanced, bioprocess, cost effective, environment friendly

In the recent times, there has been considerable focus on transition to renewable energy sources and on carbon capture and storage (CCS) of CO_2_. Hence biomass derived alternative energy products and chemicals have gained attention worldwide. The area of biofuel research is very widespread and is evolving rapidly in various aspects in order to meet ever-expanding automobile industries. Various advanced biofuels such as aviation fuels, syngas, biohydrogen, are being implemented recently and novel concepts are being executed for in-depth study/engineering of metabolic pathways or for discovery of novel bioprocesses. Recently biofuel research is geared up to establish the sustainable and economical biorefineries via concomitant production of biofuels, and value added biochemicals such as pigments/nutraceuticals (Singh et al., 2016). Environmental concerns on waste management may be ameliorated by utilizing the massive agricultural and industrial waste for the cultivation of hyperproducer oleaginous microorganisms that grow on a wide variety of sugars derived from waste hydrolysates (Singh et al., 2018; Sinha S. et al., 2020; Sinha et al., 2021). There has been a keen interest in applying versatile microbes for generation of renewable energy fuels from the biomass and biological wastes, not only because of the versatile nature of microbes for producing biofuels and other valuable products, but also due to their ability to use waste as their source of energy and nutrition (Kumar and Kumar, 2017). Using microbes also saves arable land which can otherwise be used for food and fodder and microbial cultivation does not depend upon weather conditions, unlike plant resources.

Advances in microbial biofuel production has been recently achieved through the use of cost-effective innovative feedstock, robust microbes, and stable bio/ nano-catalyst systems (Verma et al., 2016; Arora et al., 2020; Singh G. et al., 2020; Singh N. et al., 2020; Sinha S. et al., 2020). Now-a-days, the bioenergy sector is not restricted to fuel production, but extends to biorefinery where other value-added products using waste/underutilized biomass are generated in order to make the overall process cost-effective (Figure 1). Researchers are now addressing the fundamental issues of escalating fuel prices and depletion of fossil fuels through implementation of cost-effective bioenergy strategies (Sharma et al., 2018a,b, 2021).

**Figure 1:**
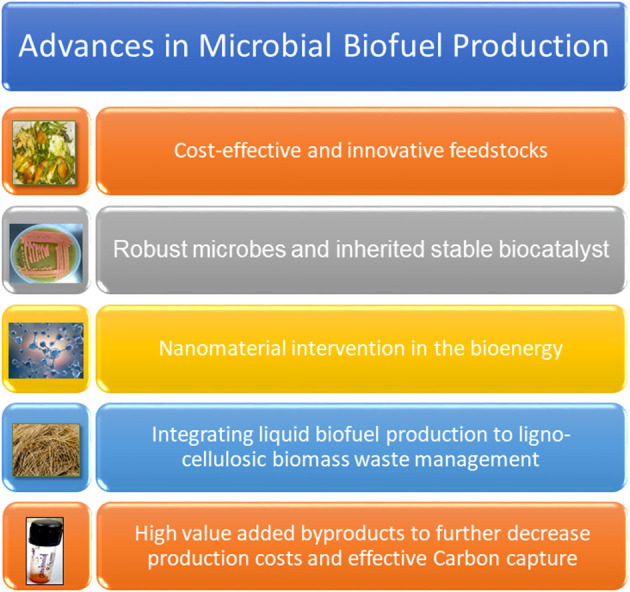
The figure showing an overview of advances made in microbial biofuel production.

Developing a robust methodology is a prerequisite requirement for quickly determining the suitability of any novel microbial strain or improvised strain toward bioenergy production (Sharma et al., 2021). For biodiesel production, oleaginous yeast strains are being explored and improved techniques are being employed for screening such strains. The researchers have investigated the insights of stability of fluorescence dye namely Nile red for estimation of lipid content in oleaginous organisms (Ramírez-Castrillón et al.) and demonstrated the occurrence of optimal fluorescence stability period between 20 and 30 min that need to be optimized with regard to chosen strain and reaction conditions. They have further reported that relative fluorescence units can be measured after the 30 min.

The cost-effective feedstock is the ideal requirement for the successful operation of any biofuel industry (Verma et al., 2016). However, the degree of ease and copious abundance of any inexpensive feedstock depends on the specific requirements of a particular geographical region. For example, Mhlongo et al. explored the potential contribution of filamentous fungi toward single cell oils for biodiesel production. However, researchers have emphasized on specific issues that restrict their potential applications and discussed the other routes for synthesis of certain biofuel/biochemicals in order to meet the requirement of ideal filamentous fungi. Another group of researchers explored the lignocellulosic biomass as the alternative feedstock for growing and harvesting of oleaginous microorganisms with the final target of biodiesel production (Chintagunta et al.). Researchers also highlighted the disadvantage associated with conventional feedstock, that should eventually be minimized through the usage of innovative lignocellulosic-based technologies or via cultivating oleaginous microbes for lipid production, detailing the steps of biodiesel production step-wise starting from lignocellulosic biomass to the actual biofuel in purified form. Advantages of cultivating oleaginous microbes for lipid production and challenges in subsequent extraction, processing via transesterification protocols and purification of biodiesel has been discussed.

Rajan et al. developed the novel approach for the prebiotic development for the production of probiotics using the underutilized hemicellulosic feedstocks. They utilized hemicellulosic hydrolysates isolated from plants such as pine, poplar and switchgrass as substrates for culturing prebiotics. The study not only revealed that prebiotic ingredients may be produced from various lignocellulosic biomass, but also correlated the viability and differential growth ability of probiotic microbes to the composition of hemicellulose-based resources.

Thus, the scope of microbial biofuel production is multifaceted that demonstrates the vastness of research and development of the bioenergy research. It may be concluded with the discussion that implementation of the innovative approaches in the design and development of cost-effective feedstocks will help to lower the escalating fuel price and ameliorate the current environmental issues across the globe in the bioenergy sector. Integrating liquid biofuel production to lignocellulosic biomass waste management has gained significant attention from circular economical aspects and environmental benefits. Adoption of such integrated processes would lead to cost-effective technologies that would generate valuable by-product(s) on one hand and regulate anthropogenic emissions of CO_2_ on the other.

## Author Contributions

All authors listed have made a substantial, direct and intellectual contribution to the work, and approved it for publication.

## Funding

This study was supported by Himachal Pradesh Council for Science, Technology & Environment (HIMCOSTE), Grant Number: STC/F(8)-6/2019(R&D 2019-20)-377 to MV.

## Conflict of Interest

The authors declare that the research was conducted in the absence of any commercial or financial relationships that could be construed as a potential conflict of interest.

## Publisher's Note

All claims expressed in this article are solely those of the authors and do not necessarily represent those of their affiliated organizations, or those of the publisher, the editors and the reviewers. Any product that may be evaluated in this article, or claim that may be made by its manufacturer, is not guaranteed or endorsed by the publisher.
